# Anaphylactic shock with methylprednisolone sodium succinate in a child with short bowel syndrome and cow’s milk allergy

**DOI:** 10.1186/s13052-017-0422-4

**Published:** 2017-11-17

**Authors:** Federica Porcaro, Maria Giovanna Paglietti, Antonella Diamanti, Francesca Petreschi, Alessandra Schiavino, Valentina Negro, Valentina Pecora, Alessandro Fiocchi, Renato Cutrera

**Affiliations:** 1 Respiratory Unit, Academic Department of Pediatrics, Bambino Gesù Children’s Hospital, IRCCS, Rome, Italy; 2 Artificial Nutrition Unit, Bambino Gesù Children’s Hospital, IRCCS, Rome, Italy; 3 Division of Allergy, Academic Department of Pediatrics, Bambino Gesù Children’s Hospital, IRCCS, Rome, Italy

**Keywords:** Anaphylaxis, Cow’s milk allergy, Children, Methyl-prednisolone sodium succinate, Short bowel syndrome

## Abstract

**Background:**

Medications with methyl-prednisolone sodium succinate containing lactose, which potentially contains traces of cow’s milk proteins (CMP), could cause allergic reactions or compromise treatment of acute allergic reactions in sensitized patients.

**Case presentation:**

We describe the unusual case of a one-year-old child affected by short bowel syndrome and history of severe cow’s milk allergy (CMA) and anaphylactic reaction due to intravenous administration of methyl-prednisolone sodium succinate (Solu-Medrol 40 mg, Pfizer). He was admitted to our hospital for severe respiratory failure and was initially treated with methyl-prednisolone (Urbason 40 mg, Sanofi Aventis), then with methyl-prednisolone sodium succinate (Solu-Medrol 40 mg, Pfizer). After the intravenous administration of second steroid, immediate anaphylaxis was recorded and treatment was stopped. Antihistamine and epinephrine were required and symptom resolution occurred.

**Conclusion:**

Children who are highly sensitive to milk may have severe allergic reactions also after exposure to CMP through a different administration route than the oral one. Patients who have food allergies need to pay particular attention to the prescription of drugs and their formulation.

## Background

Cow’s milk allergy (CMA) is one of the most common food allergies that affects up to 3.8% of children less than 5 years of age [1]. According to the European Anaphylaxis Registry, cow’s milk is one of the major elicitor food of anaphylaxis in children and adolescents [2]. Patients affected by short bowel syndrome (SBS) seem to be predisposed to develop CMA [3].

Due to atopic state and/or bronchial hyperreactivity, some small children may have recurrent episodes of acute wheezing during respiratory infections for which corticosteroids are commonly used. International guidelines such as the Global Initiative for Asthma (GINA) [4] and those by the British Thoracic Society [5] recommend the early use of systemic corticosteroids (prednisone, prednisolone, methyl-prednisolone, hydrocortisone) in acute asthma as it has been shown to reduce hospital admission rate, length of hospitalization and relapse rate after discharge.

In the present paper, we describe the case of a one-year-old child affected by SBS with history of severe CMA and anaphylactic reaction due to intravenous administration of methyl-prednisolone sodium succinate (Solu-Medrol 40 mg, Pfizer) given for the treatment of viral respiratory illness.

## Case presentation

A one-year-old male patient presented with fever, productive cough and dyspnea. On clinical examination he had pale face, nasal flaring and intercostal retraction. Physical examination of his chest revealed widespread crackles and wheezing and radiographic findings were suggestive of pneumonia. Laboratory data were not significant with the exception of nasopharyngeal aspirate that was positive for Respiratory Syncytial Virus A by PCR.

The child was affected by a polymalformative syndrome and his past medical history included necrotizing enterocolits and extended resection of affected intestinal tract. Due to short residual intestinal length after resection, he developed SBS that required long-term parenteral nutrition combined with enteral nutrition administered by gastrostomy tube. At four months of life, he experienced anaphylaxis due to cow’s milk proteins administered through gastrostomy; sensitization to CMP was confirmed by specific IgEs levels that were positive for α-lactalbumin (13.5 kU/L), β -lactoglobulin (91.9 kU/L) and casein (>100 kU/L). Avoidance of milk and dairy products was recommended and enteral nutrition with extensively hydrolyzed milk formula was suggested.

Due to the respiratory effort, the comorbidity and the precarious clinical balance during the hospitalization, antibiotic therapy with piperacillin/tazobactam was administered despite viral infection. Moreover, because of the severity of bronchospasm in the context of acute respiratory exacerbation due to viral infection, lactose free methyl-prednisolone (Urbason 40 mg, Sanofi-Aventis) was added to therapy.

After two days of therapy without any side effect, the patient was given methyl-prednisolone sodium succinate (Solu-Medrol 40 mg, Pfizer) instead of Urbason because of a temporary lack of availability.

Within few minutes of the first intravenous administration of steroid therapy, the patient presented with wheezing and generalized urticaria. Symptoms resolved after parenteral administration of antihistamine.

Because steroid administration was preceded by antibiotic therapy, an allergic reaction to piperacillin/tazobactam was suspected and antibiotic was discontinued. Steroid treatment was maintained unchanged and anaphylaxis with giant urticaria, eyelid edema, tightened laryngospasm and severe dyspnea occurred within few minutes of the second intravenous administration of 10 mg of methyl-prednisolone sodium succinate. Steroid therapy was stopped and parenteral antihistamine and epinephrine were given with prompt resolution of symptoms.

Total IgE and serum-specific IgE antibody measured with the Phadia CAP RAST system, Phadia diagnostics (the lower limit of detection was 0.35 UA/mL and the upper limit of detection was 100 UA/mL) were done during the hospitalization with increased values of total IgE (687 kU/L), α-lactalbumin IgE (22.3 kU/L), β -lactoglobulin IgE (92.7 kU/L) and casein IgE (>100 kU/L).

On the basis of the clear clinical history and laboratory findings, we hypothesized that Solu-Medrol 40 mg was the cause of the allergic reaction and the previous antibiotic and steroid therapy were restarted without side effects. Diagnostic drug challenge with lactose containing preparation was not performed due to the recent history of severe systemic reaction. Nevertheless, after 4 weeks of suspected reaction, the above hypothesis was confirmed with skin prick test and intradermal test with pure methyl-prednisolone sodium succinate used respectively at the concentration of 20 mg/ml and 2 mg/ml [6]: the tests were positive for formulation of 40 mg and negative for formulation of 125 mg. The presence of potentially allergenic milk proteins in ten different batches of the implicated product (Solu-Medrol 40 mg, Pfizer) was assessed by SDS-PAGE and Immunoblotting methods. We detected traces of milk proteins in samples from all ten batches tested, confirming our hypothesis of milk allergen contamination.

## Discussion

Few papers show that CMP may be present in certain steroid preparations. Allergic reactions to intravenous methyl-prednisolone sodium succinate (Solu-Medrol 40 mg) in allergic cow’s milk patients have already been described in the pediatric population [7–9]. Intravenous Solu-Medrol has five parenteral formulations, but only 40 mg formulation contains lactose of bovine origin as an excipient as it is indicated in the drug technical document. Even if this disaccharide sugar is relatively safe in affected CMA patients, lactose in Solu-Medrol 40 mg vials contains traces of CMP. This explains the allergic reaction to this steroid formulation and its immediate evolution due to parenteral administration route in our case. Furthermore, our patient had a SBS as underlying condition predisposing him to CMA. SBS patients have higher risk for CMA than the general population [3], due to some specific pathophysiological characteristics. These patients have low digestive capacity due to incomplete peptic digestion during early life, so protein remnants of the diet could act as allergens. This concept is also underpinned by studies on the use of anti-acid drugs that have clearly linked the impairment of gastric function with sensitization against oral proteins and drugs [10].

Therefore, when a patient with severe cow’s milk allergy needs drug therapy, only a preparation entirely free of lactose should be used, especially when it is intravenously injected. Moreover, in case of change of formulation, it is necessary to verify if any excipient contains any allergen.

With our case report, we want to emphasize that a lactose-free diet is not justified in children with cow’s milk allergy and that only foods or medications with lactose containing traces of CMP must be avoided in this group of patients.

It would be desirable that drug companies ensure that the administration of such drugs is devoid of residual CMP in all the formulations. Along these lines, the European Medicine Agency (EMA) has recently started a review of acute allergic reactions associated with injectable medicines which contain lactose from cow’s milk causing an allergic reaction in sensitized patients [http://www.ema.europa.eu/docs/en_GB/document_library/Referrals_document/Lactose_of_bovine_origin_31/Recommendation_provided_by_Pharmacovigilance_Risk_Assessment_Committee/WC500230921]. The review of these cases is aimed to evaluate the measures that reduce the risk of severe allergic reaction.

There is no level of cow’s milk proteins considered safe for these drugs when used to treat severe allergic reactions. Because of methyl-prednisolone is used for the treatment of severe allergic reactions in an emergency setting where details of the patients’ allergy history may not always be investigated, it is confirmed that the removal of CMP from the preparation is the most effective way of minimising any risks [http://www.ema.europa.eu/ema/index.jsp?curl=pages/news_and_events/news/2017/07/news_detail_002790.jsp&mid=WC0b01ac058004d5c1].

We hope that our case can be contributory to define the necessary measures reducing the risk of anaphylaxis also in special population.

## Conclusions

The effective management of food allergy includes avoidance of food allergen(s) and instruction for the use of self injection epinephrine. Nevertheless patients and their parents must be aware that other administration routes of allergen can cause serious reactions. Physicians must instruct patients and their parents more thoroughly about their allergies, above all specifying the relevant names of all allergens to which they are sensitized. Much attention must also be paid to prescribing drugs potentially containing CMP in cow’s milk sensitized patients.

## Abbreviations

CMA: Cow’s milk allergy
CMP: Cow’s milk protein
EMA: European Medicine Agency
GINA: Global Initiative for Asthma
SBS: Short bowel syndrome
